# Spermiogenesis in *Caenorhabditis elegans*: An Excellent Model to Explore the Molecular Basis for Sperm Activation

**DOI:** 10.3390/biom13040657

**Published:** 2023-04-07

**Authors:** Yoshihiro Shimada, Nana Kanazawa-Takino, Hitoshi Nishimura

**Affiliations:** Department of Life Science, Faculty of Science and Engineering, Setsunan University, Osaka 572-8508, Japan

**Keywords:** spermiogenesis, sperm activation, acrosome reaction, compound, *C. elegans*, mouse

## Abstract

*C. elegans* spermiogenesis converts non-motile spermatids into motile, fertilization-competent spermatozoa. Two major events include the building of a pseudopod required for motility and fusion of membranous organelles (MOs)—intracellular secretory vesicles—with the spermatid plasma membrane required for the proper distribution of sperm molecules in mature spermatozoa. The mouse sperm acrosome reaction—a sperm activation event occurring during capacitation—is similar to MO fusion in terms of cytological features and biological significance. Moreover, *C. elegans fer-1* and mouse *Fer1l5*, both encoding members of the ferlin family, are indispensable for MO fusion and acrosome reaction, respectively. Genetics-based studies have identified many *C. elegans* genes involved in spermiogenesis pathways; however, it is unclear whether mouse orthologs of these genes are involved in the acrosome reaction. One significant advantage of using *C. elegans* for studying sperm activation is the availability of in vitro spermiogenesis, which enables combining pharmacology and genetics for the assay. If certain drugs can activate both *C. elegans* and mouse spermatozoa, these drugs would be useful probes to explore the mechanism underlying sperm activation in these two species. By analyzing *C. elegans* mutants whose spermatids are insensitive to the drugs, genes functionally relevant to the drugs’ effects can be identified.

## 1. Introduction

During sperm activation, a prerequisite for spermatozoa to fertilize oocytes [1,2,3], spermatozoa undergo cytological and biochemical alterations resulting in the acquisition of fertilization ability [1,2,3]. However, the molecular basis for sperm activation is largely unknown in most animal species. For instance, how sperm activation is triggered and regulated and whether sperm activation mechanisms are conserved during evolution remain unclarified.

The nematode *Caenorhabditis elegans* has been used to investigate male germline functions, such as production of spermatids via meiosis, formation of fertile spermatozoa from the spermatids via spermiogenesis, and fertilization of oocytes by the spermatozoa [4,5,6,7,8]. Because spermiogenesis in *C. elegans* readily occurs in vitro and it has abundant genetic resources, *C. elegans* is an excellent model to elucidate the mechanism for sperm activation [4,5,6,7,8]; many *C. elegans* genes involved in spermiogenesis pathways have been identified by genetics-based studies. Moreover, drugs capable of triggering or blocking spermiogenesis are available. Targets of these drugs in spermatids can be examined by analyzing *C. elegans* mutants in which the drugs are less or more potent.

Here, we review the recent findings on the regulation of *C. elegans* spermiogenesis, including sperm activation. We also discuss that *C. elegans* and mice might share a common molecular basis for sperm activation. Unlike in mammals, the term “spermiogenesis” has often been used interchangeably with “sperm activation” in *C. elegans*, owing to that spermatozoa capable of fertilizing oocytes are formed during spermiogenesis (for details, see Section 2.1). In this review, we consistently used “sperm activation” as an event for spermatozoa to acquire fertility, enabling to compare its process and mechanism between *C. elegans* and mice.

## 2. Overview of *C. elegans* Spermiogenesis

### 2.1. Male Reproductive Processes in Mice and C. elegans

**Figure 1:**
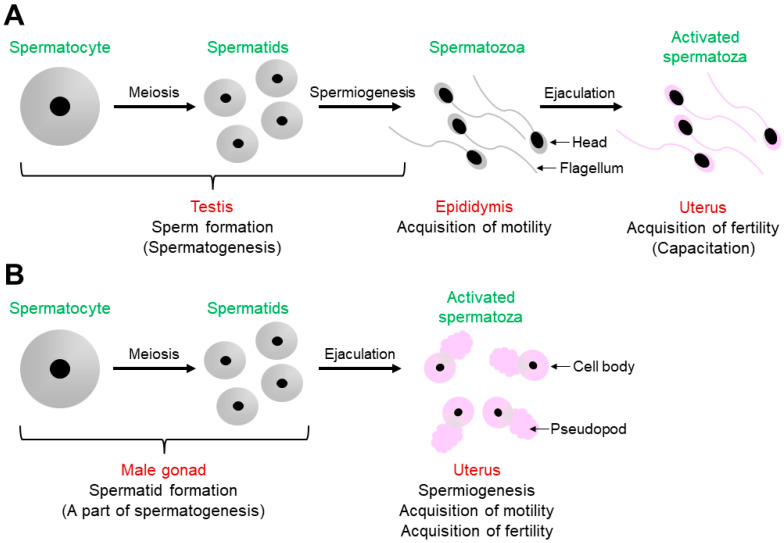
Comparison of sperm formation and activation in mice and *C. elegans*. (**A**) In the mouse testis, spermatozoa are produced during spermatogenesis. The testicular spermatozoa acquire their motility during transition in the epididymis. After ejaculation, the epididymal spermatozoa undergo capacitation to gain the ability to fertilize oocytes. (**B**) Meanwhile, in the *C. elegans* male gonad, round spermatids are produced via meiosis; however, spermatogenesis is arrested at this stage. After mating, in the uterus of hermaphrodites, spermatogenesis resumes, and ejaculated spermatids are then activated presumably by TRY-5, which is contained in ejaculated seminal fluids. This process is called spermiogenesis, during which spermatozoa acquire motility and fertility. Pink-colored spermatozoa indicate that they are fertilization-competent.

As shown in Figure 1A, mouse spermatogenesis occurs in the testis to produce spermatids from spermatocytes via meiosis, and the resulting spermatids are transformed into spermatozoa in a process called spermiogenesis [1,9]. Testicular spermatozoa become motile during the transition from the caput to the cauda epididymis [1]. After ejaculation, spermatozoa ascend from the uterus into the oviduct to meet and fertilize the oocytes. Before fertilization in the oviduct, spermatozoa undergo capacitation in the uterus, during which they become fertilization-competent [1,2,3].

Some of the *C. elegans* male germline functions are shown in Figure 1B [4,5]. In the male gonad, which is functionally equivalent to the mouse testis, a spermatocyte is meiotically divided into spermatids; however, spermatogenesis is halted at this stage. After mating, spermatids are ejaculated into the uterus with seminal fluids. Spermatogenesis then resumes by a spermatid-activating factor (SAF) contained in the fluids [10], which is presumably the serine protease TRY-5 [4,5,6,7,8,9,11]. In other words, *C. elegans* spermiogenesis in male-derived spermatids occurs in the uterus of hermaphrodites, not in the male gonad. Moreover, unlike in mouse spermiogenesis, spermatids are transformed into spermatozoa with fertility in *C. elegans* spermiogenesis [4,5,6,7,8].

### 2.2. Acrosome Reaction in Mice and Membranous Organelle Fusion in C. elegans

As described in Section 2.1, ejaculated mouse spermatozoa acquire fertility during capacitation. This phenomenon includes the acrosome reaction, tyrosine phosphorylation of sperm proteins, acquisition of a different pattern of sperm motility (hyperactivation), and dynamic alteration of the plasma membrane (PM) status of the sperm head [1,2,3].

**Figure 2:**
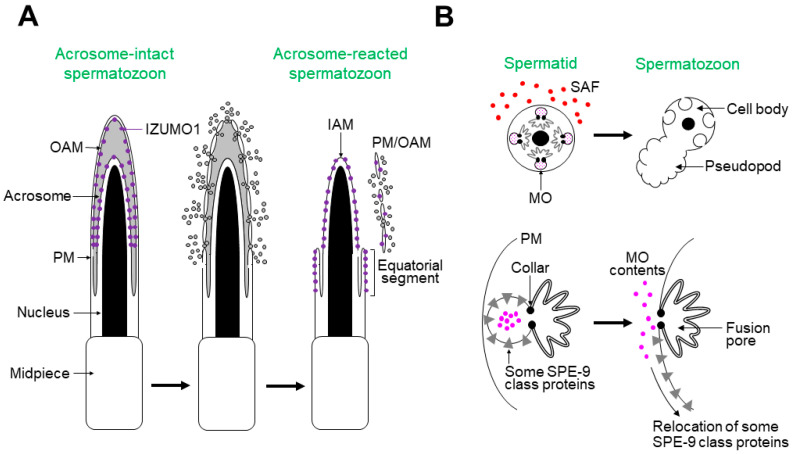
The acrosome reaction in mice and MO fusion in *C. elegans*. (**A**) The acrosome reaction is a sperm activation event during capacitation. After initiation of the acrosome reaction, the PM and OAM fuse at multiple sites, resulting in the extracellular release of acrosomal contents (gray-colored circles). IZUMO1 (purple-colored circles)—a sperm protein essential for gamete fusion—relocates to the surface of the equatorial segment, where the spermatozoa bind to and fuse with the oocyte PM. In this panel, the flagellum is omitted. (**B**) (**Upper panel**) A *C. elegans* spermatid intracellularly contains numerous MOs. After stimulation with in vivo or in vitro SAFs (red-colored circles), a pseudopod extends from the cell, forming a motile amoeboid spermatozoon. (**Lower panel**) Simultaneously, each MO fuses with the spermatid PM, and MO contents (pink-colored circles) are released extracellularly. The MO collars leave a permanent fusion pore. During acrosome reaction, some SPE-9 class proteins, which are essential for fertilization, relocate onto the pseudopod or entire spermatozoon. This illustration is a modification of a figure in [9].

Figure 2A shows the process of the acrosome reaction in mouse spermatozoa [1,2,3,9]. After an unknown physiological activator(s) triggers the acrosome reaction, the PM and outer acrosomal membrane (OAM) fuse at multiple points on the sperm head, releasing acrosomal contents extracellularly. The acrosome-reacted spermatozoa have the following features: (1) the inner acrosomal membrane (IAM) is exposed on the sperm head; (2) the equatorial segment is newly formed, where the spermatozoa bind to and fuse with the oocyte PM; (3) IZUMO1, a sperm protein essential for gamete fusion, partly relocates to the equatorial segment surface [12].

**Figure 3:**
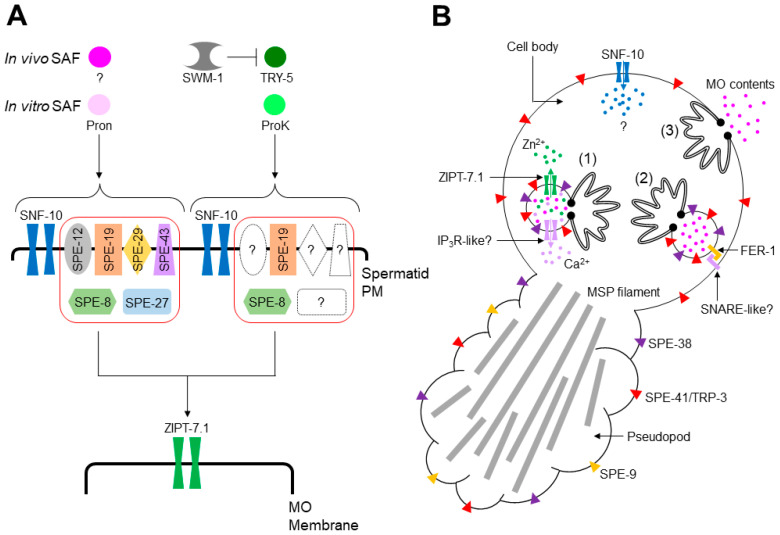
Summary of *C. elegans* spermiogenesis. (**A**) A proposed model of spermiogenesis pathways. One pathway is dependent on SPE-8 class proteins (enclosed in red box) and SNF-10 and is activated by in vivo SAF (hSAF, dark pink-colored circle) or in vitro SAF (the bacterial protease mixture Pronase (Pron), light pink-colored circle). Another pathway also depends on SNF-10 but only partially requires SPE-8 class proteins. Broken-lined symbols represent SPE-8 class proteins whose functions are not essential for this pathway. The SPE-8 class-independent pathway is possibly activated by the male-produced serine protease TRY-5 (in vivo SAF (mSAF), dark green-colored circle) or bacterial serine protease Proteinase K (ProK; in vitro SAF, light green-colored circle). In the gonads, TRY-5 and SWM-1 form a protein complex to prevent ectopic spermiogenesis. SWM-1 has two trypsin inhibitor-like domains, implying that an additional unknown serine protease might act as in vivo SAF. Two pathways indicated here would merge at a certain point, and ZIPT-7.1 presumably functions downstream of that point. (**B**) Pseudopod extension and MO fusion during spermiogenesis. The formed pseudopod contains major sperm protein (MSP) filaments (light gray-colored thick bars), which grant motility via cytoskeleton assembly and disassembly. Steps (1–3) represent the events during MO fusion. In Step (1), some colored circles show MO contents such as Zn^2+^ (green), Ca^2+^ (purple), and other soluble contents (pink). The blue-colored circle represents an unknown substance imported into spermatids via SNF-10. It is currently unclear where and how Ca^2+^ is released into the cytoplasm, but an inositol 1,4,5-trisphosphate receptor (IP_3_R)-like protein(s) might reside on the MO membrane. In Step (2), FER-1 might associate with an unknown SNARE protein, mediating the fusion between the MO membrane and spermatid PM. After completion of MO fusion, represented by Step (3), MO contents are released extracellularly, and SPE-9 on the spermatid PM and SPE-38 and SPE-41/TRP-3 on the MO membrane are relocated to the surfaces of the pseudopod (SPE-9 and SPE-38) or entire spermatozoon (SPE-41/TRP-3). The pseudopod is where a spermatozoon interacts with an oocyte.

Sperm activation in *C. elegans* is represented by membranous organelle (MO) fusion, which occurs during spermiogenesis (Figure 2B) [4,5,6,7,8,9]. Numerous MOs, specialized secretory vesicles, are contained in a spermatid and fuse with the spermatid PM upon stimulation with in vitro or in vivo SAFs. The MO contents are then released extracellularly. *C. elegans* SPE-9 class proteins are indispensable for fertilization, some of which relocate from the MO membrane to the surfaces of the pseudopod or entire spermatozoon (for details, see Figure 3B). Notably, the pseudopod is the site where the spermatozoa interact with oocytes; hence, MO fusion is similar to the acrosome reaction regarding cytological features and biological significance.

### 2.3. Spermiogenesis Pathways in C. elegans

Previous genetics-based studies have provided important clues for solving the molecular basis of *C. elegans* spermiogenesis. This animal has two sexes—hermaphrodites and males—producing spermatids. Hermaphrodite- and male-derived spermatids seemingly undergo SPE-8 class-dependent and -independent pathways [4,5,6,7,8].

*spe* (spermatogenesis-defective) genes play important roles in *C. elegans* male germline functions, such as meiosis, spermiogenesis, and fertilization, and many of these genes are specifically or predominantly expressed in the male germline (Table 1) [4,5]. One category of *spe* genes is the *spe-8* class, whose members include *spe-8*, *spe-12*, *spe-19*, *spe-27*, *spe-29*, and *spe-43* (Table 1). Mutants deficient in one of these genes exhibit similar phenotypes, i.e., hermaphrodites are self-sterile, and males are cross-fertile, suggesting that the SPE-8 class-dependent pathway is activated by an unknown hermaphrodite-produced SAF (hSAF). Moreover, the bacterial protease mixture Pronase (Pron) might trigger the pathway as an in vitro SAF (Figure 3A), since Pron can activate wild-type spermatids, but not those with *spe-8* class mutations [4,5].

The SPE-8 class-independent pathway is likely to be male-dependent and activated by TRY-5 as a male-produced SAF (mSAF; Figure 3A) [11]. SWM-1, a protein containing two trypsin inhibitor-like domains [28], may form a protein complex with TRY-5, blocking ectopic spermiogenesis in male gonads (Figure 3A) [11,28]. Although in vitro SAFs that can activate this pathway have not been identified thus far, the bacterial serine protease Proteinase K (ProK) was recently revealed as a candidate [34]. Unlike in vitro stimulation with Pron, that with ProK completely activated *spe-12*, *spe-27*, and *spe-27* mutant spermatids and partially activated *spe-8* and *spe-19* mutant cells (Figure 3A) [34]. However, whether TRY-5 activates the ProK pathway remains unclear.

Several genes may act in both the Pron and ProK pathways. As the *snf-10* gene is disrupted in *C. elegans*, hermaphrodites are self-fertile, while males are cross-sterile [13], suggesting that SNF-10 is involved in the male-dependent spermiogenesis pathway. Neither Pron nor ProK can activate *snf-10* mutant spermatids, despite the self-fertility of *snf-10* mutant hermaphrodites [13,34], indicating that SNF-10 functions in the Pron and ProK pathways (Figure 3A) and that the hermaphrodite-dependent spermiogenesis pathway might not equal the Pron pathway. *zipt-7.1* is required for the fertility of hermaphrodites and males [27]. Furthermore, Pron and ProK failed to activate *zipt-7.1* mutant spermatids [27,34], indicating that ZIPT-7.1 functions in the Pron and ProK pathways.

Certain alleles in *spe-4* [16], *spe-6* [17], *spe-46* [25] and *spe-47* [26] suppress mutations in *spe-8* class genes, partially rescuing the self-sterility of *spe-8* class mutant hermaphrodites, and these suppressor mutations also cause premature spermiogenesis (Table 1). Spermatid proteins encoded by these *spe* genes seemingly exhibit “brake”-like functions in spermiogenesis, preventing premature spermatid activation until one or some of the SPE-8 class proteins yield a signal(s) that triggers spermiogenesis. In other words, *spe-4*, *spe-6*, *spe-46*, and *spe-47* act downstream of *spe-8* class genes. If the “brake” model is correct, how SPE-47 acts as a “brake” despite its absence in spermatids warrants investigation [26].

Several lines of information are available regarding how the genes listed in Table 1 are functionally interrelated. First, when mitogen-activated protein kinases (MAPKs) are activated in vitro in spermatids [34,35], spermiogenesis is induced in *snf-10* mutant spermatids but not in *zipt-7.1* mutant cells, suggesting that SNF-10 presumably resides upstream of ZIPT-7.1 in the Pron and ProK pathways [34]. This does not conflict with the finding that *zipt-7.1* is located genetically downstream of *spe-6* [27]. Second, the data obtained from yeast two-hybrid screening imply that ZIPT-7.1 physically binds to SPE-4 [27] and SPE-43 to SPE-4 and SPE-29 [24]. However, the complete molecular picture of the *C. elegans* spermiogenesis pathways remains largely unknown. Identification of physiological SAFs and their direct targets should thus be prioritized.

### 2.4. Molecular Annotations of Cellular Reactions Occurring during C. elegans Spermiogenesis

After stimulation of either spermiogenesis pathways, *C. elegans* spermatids undergo various cellular reactions to produce fertilization-competent spermatozoa. As spermatids are transcriptionally silent cells, the acquisition of sperm motility and fertility is unaccompanied by de novo protein synthesis. Figure 3B shows the cellular reactions that occur during spermiogenesis. To transform quiescent, round spermatids into motile, amoeboid spermatozoa, pseudopods extend from the spermatids. Sperm motility is endowed by the assembly and disassembly of major sperm protein (MSP) filaments contained in the pseudopod, and sperm cytoskeleton dynamics are regulated by a network of kinases and phosphatases [36,37].

Sperm motility is necessary but not sufficient to fertilize oocytes. In *C. elegans*, spermatids need intracellular mediators that allow spermiogenesis to proceed to achieve full fertility. The *snf-10* gene encodes a member of the solute carrier protein 6 (SLC6) family that transports amino acids or neurotransmitters in a Na^+^-dependent manner [38], suggesting that in the early phase of spermiogenesis (Figure 3A), SNF-10 on the spermatid PM imports unknown substance(s) into the cytoplasm (Figure 3B). Zn^2+^ may also act as an intracellular mediator of spermiogenesis [27,39,40]. Zn^2+^ is contained within MOs, where the zinc transporter ZIPT-7.1 localizes (Figure 3B), suggesting that Zn^2+^ is released into the cytoplasm via ZIPT-7.1 in the middle-to-late phase of spermiogenesis (Figure 3A). Ca^2+^ mediates diverse cellular responses [41,42]. In spermatids with chelated intracellular Ca^2+^, MO fusion is blocked after in vitro stimulation with SAF [31]. In contrast, the depletion of extracellular Ca^2+^ does not affect MO fusion [43]. These findings suggest that intracellular Ca^2+^ is pivotal in spermiogenesis (Figure 3B). Moreover, compounds antagonistic to phospholipase C and inositol 1,4,5-trisphosphate receptors (IP_3_Rs) block in vitro SAF-triggered spermiogenesis, implying the involvement of Ca^2+^ signaling during spermiogenesis [35]. However, the source(s) of intracellular Ca^2+^ in *C. elegans* spermatids is unknown, similar to that in mouse spermatozoa, although the nucleus, mitochondria, and MOs (secretory vesicles) are candidates for intracellular Ca^2+^ stores [44].

Steps (1–3) in Figure 3B show the proposed process of MO fusion. In resting spermatids, the interior of MOs is acidified via SPE-5, a B subunit of vacuolar H^+^-ATPase (V-ATPase) [45]. SPE-38 [46] and SPE-41/TRP-3 [47], members belonging to the SPE-9 class, are localized on the MO membrane, whereas SPE-9, another SPE-9 class protein, resides on the spermatid PM [48]. After spermiogenesis is initiated, intracellular mediators, such as Zn^2+^ and presumably Ca^2+^, are delivered into the cytoplasm from the inside of MOs (Step (1), Figure 3B).

Step (2) shows the ongoing status of MO fusion (Figure 3B). The pH inside MOs might increase following negative regulation of V-ATPase, as during the acrosome reaction [49]. *C. elegans fer-1* encodes a member of the ferlin family that carries C2 domains on the MO membrane. This domain generally mediates Ca^2+^-dependent lipid-processing events [31]. Mutations in this gene cause a defect in MO fusion, and *fer-1* mutant spermatids become hypersensitive to intracellular Ca^2+^ depletion [31]. Thus, FER-1 is likely indispensable for Ca^2+^-dependent MO fusion. The mouse *Fer1l5* gene is specifically expressed in the testis and encodes an ortholog of *C. elegans fer-1* [50]. Spermatozoa from male mice deficient in *fer1l5* failed to undergo the acrosome reaction, and the Fer1l5 protein might bind to the syntaxin 2 protein [50], which belongs to the SNARE family [51]. Since SNAREs are involved in various membrane fusions, the binding between Fer1l5 and syntaxin 2 emphasizes the importance of Fer1l5 in the acrosome reaction. These findings imply that MO fusion and acrosome reaction may share a ferlin-based fusogenic machinery. In particular, given that Fer1l5 forms a protein complex with syntaxin 2 at least in vitro [50], a SNARE protein(s) on the spermatid PM may be a *trans*-binding partner of FER-1 on the MO membrane (Step (2), Figure 3B).

Following MO fusion with the spermatid PM, MO contents are released extracellularly (Step (3), Figure 3B); however, their roles are currently unclear. Regarding the localization of SPE-9 class proteins, SPE-9 [48] and SPE-38 [46] are translocated onto the pseudopod surface and SPE-41/TRP-3 onto the entire surface of the spermatozoa [47] (Step (3), Figure 3B). These translocations of SPE-9 class proteins presumably enable spermatozoa to fertilize oocytes, as fertilization occurs via contact between the spermatozoon pseudopod and an oocyte. A recent finding demonstrated that Na^+^/K^+^-ATPase (NKA), composed of CATP-4 and NKB-2 subunits, plays crucial roles in spermiogenesis and sperm motility [52]; spermatids from males lacking *catp-4* or *nkb-2* failed to extend normal-length pseudopods in in vitro spermiogenesis, presumably leading to a defect in sperm motility. Relevant to these phenotypes, NKA on the spermatid PM is translocated onto the fusion pore of spermatozoa during spermiogenesis. The translocation of NKA depends on the transport of cholesterol from the spermatid PM to MOs via membrane contact sites (MCSs); the phosphoinositide 5-phosphatase CIL-1 (see Section 3.1) and the phosphoinositide 4-phosphatase SAC-1 may contribute to cholesterol transport.

## 3. Pharmacological Approach to the Mechanism Underlying *C. elegans* Spermiogenesis

**Figure 4:**
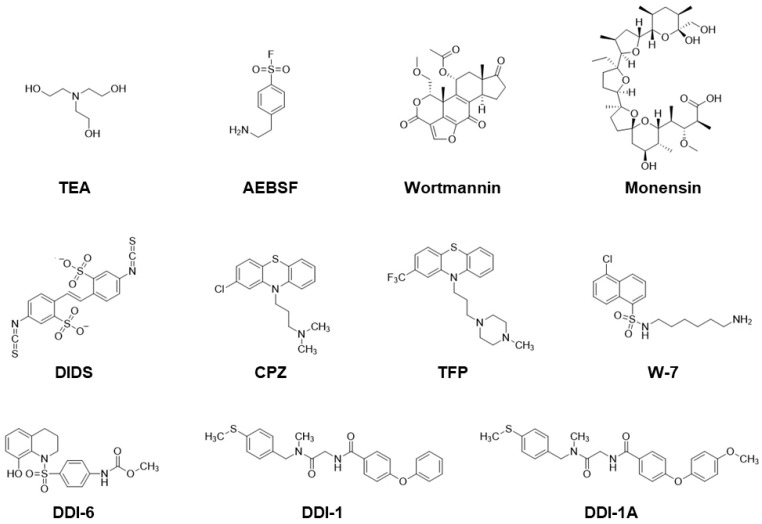
Chemical structures of the compounds that trigger or block spermiogenesis. For details, see Section 3.1 and Section 3.2.

The distinctive features of *C. elegans* spermatogenesis are: (1) spermatogenesis is arrested after meiosis is completed; (2) certain male germline functions, such as meiosis and spermiogenesis, can readily occur in a chemically defined medium. These features enable identifying physiological SAFs that trigger spermiogenesis, as well as spermatid proteins involved in spermiogenesis pathways. Moreover, pharmacological analysis can be applied in vitro. Some studies have used various compounds that activate or block spermiogenesis, providing valuable information for understanding *C. elegans* spermiogenesis regulation. Therefore, in addition to abundant genetic resources, the availability of pharmacological tools would increase the chances of identifying spermiogenesis-related factors. Figure 4 shows the chemical structures of the drugs that trigger *C. elegans* spermiogenesis or that selectively block the extension of pseudopods from spermatids.

### 3.1. Compounds Triggering Spermiogenesis

- Triethanolamine (TEA): TEA is a weak base that increases intracellular pH in spermatids [53]. Since it can activate *spe-8* and *spe-12* [19] but not *fer-1* mutant spermatids [31], TEA might trigger spermiogenesis in a FER-1-dependent manner by affecting downstream SPE-8 class proteins. The half-maximal effective concentration (EC_50_) is approximately 10 mM at pH 7.8 [53].
- MAPK activators: 4-(2-Aminoethyl)benzenesulfonyl fluoride (AEBSF), an irreversible inhibitor of serine proteases, also acts as an activator of MAPKs [54]. AEBSF induces spermiogenesis in *spe-8* class and *snf-10* mutant spermatids but not in *zipt-7.1* mutant cells [34]. AEBSF presumably affects the downstream of SPE-8 class proteins and SNF-10 and the upstream of ZIPT-7.1. The concentration employed for the assay (conc.) was 5 mM at pH 7.4 [34] or 0.5–20 mM at pH 7.0 [35]. Anisomycin (not shown in Figure 4) is a potent protein synthesis inhibitor and activator of c-Jun N-terminal kinase (JNK) [55], a member of the MAPK family. Like AEBSF, anisomycin can trigger spermiogenesis in spermatids, presumably via activation of JNK [35]. Conc. = 1 or 1.6 mM at pH 7.4 [35].
- Wortmannin: Phosphatidylinositol 3-kinase is blocked by wortmannin. When wild-type spermatids, but not mutant cells lacking *cil-1*, are treated with wortmannin, spermiogenesis is initiated [56]. Conc. = 100 nM at pH 7.8 [56].
- Monensin: This is a Na^+^- and K^+^-transporting ionophore. The extracellular Na^+^ and K^+^ concentrations affect in vitro spermiogenesis [53]. Similar to that with TEA, activation of wild-type spermatids with monensin [43] is accompanied by an increase in intracellular pH [53], and triggering spermiogenesis can also be observed in *spe-8* and *spe-12* mutants [19]. EC_50_ ≈ 20 nM at pH 7.8 [53].
- 4,4′-Diisothiocyanatostilbene-2,2′-disulfonic acid (DIDS): This agent likely blocks Clir, an inward-rectifying chloride channel on the *C. elegans* spermatid PM [57]. DIDS can activate the wild-type but not *spe-8* class mutant spermatids [57], suggesting that DIDS-induced spermiogenesis is linked to the SPE-8 class-dependent pathway. EC_50_ ≈ 500 µM at pH 7.8 [57].
- Calmodulin inhibitors: Chlorpromazine (CPZ), trifluoperazine (TFZ), and *N*-(6-aminohexyl)-5-chloro-1-naphthalenesulfonamide (W-7) belong to this category. These compounds trigger spermiogenesis at similar concentrations (EC_50_ ≈ 20 µM at pH 7.8) [19]; however, whether calmodulin is involved in activating spermatids remains to be clarified.
- DDI-6: This compound was screened as an in vitro SAF of *C. elegans* spermiogenesis from the Core Library, provided by the Drug Discovery Initiative (DDI), Tokyo, Japan [58]. DDI-6 also activates the acrosome reaction in mouse spermatozoa [58]. How DDI-6 triggers spermiogenesis in *C. elegans* spermatids and the acrosome reaction in mouse spermatozoa remains unknown. Conc. = 100 µM at pH 7.4 [58].

### 3.2. Compounds Blocking Pseudopod Extension but Not MO Fusion

- DDI-1 and its analogs: As spermatids are treated with Pron or ProK in the presence of DDI-1, the cells can undergo MO fusion but fail to extend pseudopods [34]. Moreover, some DDI-1 analogs (DDI-1A, DDI-1C, and DDI-1H; the two latter drugs are not shown in Figure 4) exhibit similar effects. These compounds block Pron-triggered pseudopod extension, but not that of ProK [34], suggesting that the original DDI-1 and DDI-1 analogs can dissect spermiogenesis pathways into the reactions of MO fusion and pseudopod extension, enabling us to focus on the mechanism of pseudopod extension during spermiogenesis. Conc. = 100 µM at pH 7.4 [34].

## 4. Conclusions and Future Perspectives

Unlike that in mammals, spermiogenesis in *C. elegans* is accompanied by acquisition of the ability to fertilize oocytes. Because MO fusion is similar to the mouse sperm acrosome reaction in terms of cytological features and biological significance, we hypothesize that these two sperm activation events share a certain molecular basis. For instance, *C. elegans* FER-1 and murine Fer1l5 are essential for fusogenic reactions between MO and the spermatid PM and between the acrosome and spermatozoon PM, respectively, implying that some proteins that activate FER-1- or Fer1l5-based fusogenic machinery might be conserved in *C. elegans* and mice. Cholesterol efflux via MCSs may be also important for sperm activation in *C. elegans* [52] and mice [59]; the acrosome is closely apposed to the spermatozoon PM in the mouse sperm head (Figure 2A), like localization of MOs beneath the *C. elegans* spermatid PM. Relevant to the cholesterol efflux, *C. elegans* [52] and murine [60] MKAs might have a common role in spermatids and spermatozoa, respectively. Moreover, RNA helicases contained in germ granules likely act during male germ cell functions in *C. elegans* and mice; GLH-1, a homolog of the germline-specific Vasa/DDX4 DEAD-box RNA helicase, represses spermatogenic expression during oogenesis in *C. elegans*, while it promotes MSP expression to drive spermiogenesis and sperm motility [61]. In mice, the mouse *Vasa* homolog gene *Mvh* is involved in proliferation and differentiation of male germ cells [62].

To identify spermiogenesis-related proteins, pharmacological and genetic studies are reasonable approaches in *C. elegans*. If certain biological phenomena can occur in in vitro systems, compounds are useful tools for examining how these events are regulated. However, any compounds may exhibit off-target activities under different conditions. Therefore, the effects of some drugs on *C. elegans* spermiogenesis (described in Section 3) might be caused by their off-target activities. To evaluate pharmacological data appropriately, genetics-based methods are also important. For instance, we isolated *C. elegans* mutants in which spermatids normally extend pseudopods via Pron-stimulation even in the presence of DDI-1 [34]. Defective genes in mutants likely disturb the inhibitory effect of DDI-1 on pseudopod extension.

The same approach can be applied to spermiogenesis-triggering drugs. As DDI-6 does, compounds capable of activating both spermatids from *C. elegans* and spermatozoa from other animal species, such as mice, presumably bind to spermatid/spermatozoon proteins that are conserved in some organisms. Identifying the targets of these compounds would clarify the common molecular basis for sperm activation in *C. elegans* and other species besides nematodes. The availability of pharmacological and genetic data for studying sperm activation is a significant advantage of using *C. elegans* as an animal model.

## Figures and Tables

**Table 1:** Genes involved in *C. elegans* spermiogenesis pathways.

Gene	LG	M/F ^1^	Encoded Protein	Sex of Mutants in Which Spermiogenesis Does Not Occur	Ref.
*snf-10*	V	15.9	SLC6 transporter (673 aa)	Male	[13]
*spe-4*	I	2.72	Presenilin-like Asp protease (465 aa)	Hermaphrodite and male	[14,15,16]
*spe-6*	III	ND	Casein kinase 1-like Ser/Thr kinase (379 aa)	Hermaphrodite and male	[17]
*spe-8*	I	2.92	Non-receptor type Tyr kinase with a SH2 domain (512 aa)	Hermaphrodite	[18,19]
*spe-12*	I	1.20	Single-pass TM protein (255 aa)	Hermaphrodite and partially male	[18,19,20]
*spe-19*	V	4.63	Single-pass TM protein (300 aa)	Hermaphrodite	[21]
*spe-27*	IV	9.32	Soluble protein (131 aa)	Hermaphrodite	[22]
*spe-29*	IV	ND	Single-pass TM protein (66 aa)	Hermaphrodite	[23]
*spe-43*	IV	3.97	Soluble protein (226 aa) Single-pass TM protein (273 aa)	Hermaphrodite	[24]
*spe-46*	I	35.8	Six-pass TM protein (290 aa)	Hermaphrodite and male	[25]
*spe-47*	I	5.94	Soluble protein with an MSP domain (380 aa)	Hermaphrodite and male	[26]
*zipt-7.1*	IV	1.00	Zinc transporter (393 aa)	Hermaphrodite and male	[27]
*try-5*	V	0.95	Ser protease (327 aa)	Male	[11]
*swm-1*	V	2.15	Soluble protein with two TIL domains (135 aa)	Male	[28]
*fer-1*	I	8.24	Ca^2+^-dependent lipid-binding protein (2034 aa)	Hermaphrodite and male	[29,30,31]

Abbreviations: LG, linkage group; SLC6, solute carrier 6; aa, amino acid; ND, not determined; SH2, src homology 2; TM, transmembrane; MSP, major sperm protein; TIL, trypsin inhibitor-like. ^1^ Ratio of gene expression in *fem-3* (male germline-specific, M) and *fem-1* (female germline-specific, F) mutant worms, calculated from data based on previous reports [32,33].

## Data Availability

All data required to evaluate the conclusions of this paper are presented in this paper.
